# Automatic Production and Preliminary PET Imaging of a New Imaging Agent [^18^F]AlF-FAPT

**DOI:** 10.3389/fonc.2021.802676

**Published:** 2022-01-06

**Authors:** JiaWen Huang, LiLan Fu, KongZhen Hu, Shun Huang, YanJiang Han, Run Lin, WanBang Xu, Ganghua Tang, Yonghui Huang

**Affiliations:** ^1^ Department of Interventional Radiology, The First Affiliated Hospital, Sun Yat-sen University, Guangzhou, China; ^2^ Department of Nuclear Medicine, Nanfang Hospital, Southern Medical University, Guangzhou, China; ^3^ Department of Traditional Chinese Medicine, Guangdong Institute for Drug Control, Guangzhou, China

**Keywords:** cancer-associated fibroblasts, PET/CT, fibroblast activation protein, [^18^F]AlF-FAPT, automatic synthesis

## Abstract

**Background:**

Fibroblast activating protein (FAP) has become an important target for cancer diagnostic imaging and targeted radiotherapy. In particular, [^18^F]FAPI-42 has been successfully applied to positron emission tomography (PET) imaging of various tumors. However, it exhibits high hepatobiliary metabolism and is thus not conducive to abdominal tumor imaging. This study reports a novel ^18^F-labeled FAP inhibitor, [^18^F]AlF-FAPT, a better FAPI imaging agent than [^18^F]FAPI-42.

**Materials and Methods:**

The precursor of [^18^F]AlF-FAPT (NOTA-FAPT) was designed and synthesized using the standard FMOC solid phase synthesis method. [^18^F]AlF-FAPT was subsequently synthesized and radiolabeled with ^18^F using the AllInOne synthesis module. Dynamic MicroPET and biodistribution studies of [^18^F]AlF-FAPT were then conducted in xenograft tumor mouse models to determine its suitability.

**Results:**

The precursors NOTA-FAPT were obtained with a chemical purity of > 95%. [^18^F]AlF-FAPT was synthesized automatically using the cassette-based module AllInOne within 40 min. The non-decay corrected radiochemical yield was 25.0 ± 5.3% (n=3). *In vivo* imaging and biodistribution studies further demonstrated that compared with [^18^F]-FAPI-42, [^18^F]AlF-FAPT had a lower hepatobiliary uptake than [^18^F]FAPI-42, which was advantageous for imaging abdominal tumors.

**Conclusion:**

[^18^F]AlF-FAPT can be synthesized automatically using a one-step method of aluminum fluoride. Collectively, [^18^F]AlF-FAPT is a better FAPI imaging agent than [^18^F]FAPI-42. This study proves the feasibility of using [^18^F]AlF-FAPT as a new radioactive tracer for PET imaging.

## Introduction

Traditional cancer diagnosis and treatment methods mainly targeted the tumor parenchyma cells. However, the tumor microenvironment and tumor stroma have increasingly become the focus of attention recently, making it possible to develop new diagnoses and treatment strategies. Cancer-associated fibroblasts (CAF) are the main components of tumor stroma (1). The difference between CAF and normal fibroblasts lies in the specific expression of fibroblast activating protein (FAP) (2, 3). The expression of FAP is usually related to poor prognosis and outcomes of cancers (4). FAP has dipeptidyl peptidase and collagenase activity, which can cleave many dipeptidyl peptidase activity substrates such as gelatin and denatured type I collagen in the matrix and participate in the degradation of extracellular matrix (ECM). It also promotes tumor cell detachment, invasion, and metastasis from the primary site (5, 6). Though it is almost nonexistent in normal tissues, it is highly expressed in cancer-associated fibroblasts (CAF) of various tumors, such as ovarian, pancreatic, and hepatocellular carcinoma (7). Therefore, it is expected to become a new tumor tracer with potential.

Recently, several positron emission tomography(PET) tracers for FAP have been successfully developed. Among them, ^68^Ga-labeled fibroblast activating protein inhibitor(FAPI) tracer exhibit a high tumor-to-organ ratio and rapid elimination in preclinical trials (8, 9). However, its short half-life limits its clinical application. In contrast, ^18^F has a longer half-life and thus has more advantages in clinical applications (10). Recently, ^18^F-labeled FAPI tracer [^18^F]FAPI-42 has been synthesized and preclinically evaluated, showing the same or even higher tumor uptake value than [^68^Ga]FAPI-04. However, it has a lower detection rate of abdominal tumors because of its excretion through the hepatobiliary pathway (11). In this study, a novel FAPI tracer linked with 1,4,7-triazacyclononane-1,4,7-triacetic acid (NOTA) was introduced to reduce hepatobiliary metabolism through the modification of FAPI pharmacophore, so as to improve the results of tumor imaging.

## Material and Methods

### Source of Chemicals and Equipment Used

All reagents and solvents were commercial products and were thus used without further purification. The General Electric PETtrace Biomedical Cyclotron (PET800, GE, USA) was used to produce [^18^F] fluoride by bombarding a high pressure [^18^O] H_2_O target with 18MeV protons. The High Performance Liquid Chromatography system (LC 20A, Shimadzu, Japan) was equipped with a UV detector (254 nm) and a C-18 column. A Thermo Fisher Science Orbitrap Fusion mass spectrometer (Thermo Fisher Scientific, USA) was used to obtain high-resolution mass spectrometry (HRMS). Automatic synthesis was carried out through the AllInOne module (Trasis, Ans, Belgium). A γ-counter (CAPRAC -R, Capintec, Inc., Ramsey, New Jersey, USA) was used to measure the radiation value of the biological distribution experiment. Small animal imaging was performed using an Inveon Micro-PET/CT scanner (Siemens, Erlangen, Germany). The chemical groups used included Polyethylene glycol (PEG), Aspartic acid group (Asp), and Glutamic acid group (Glu).

### Synthesis of Compounds

**Scheme 1:**
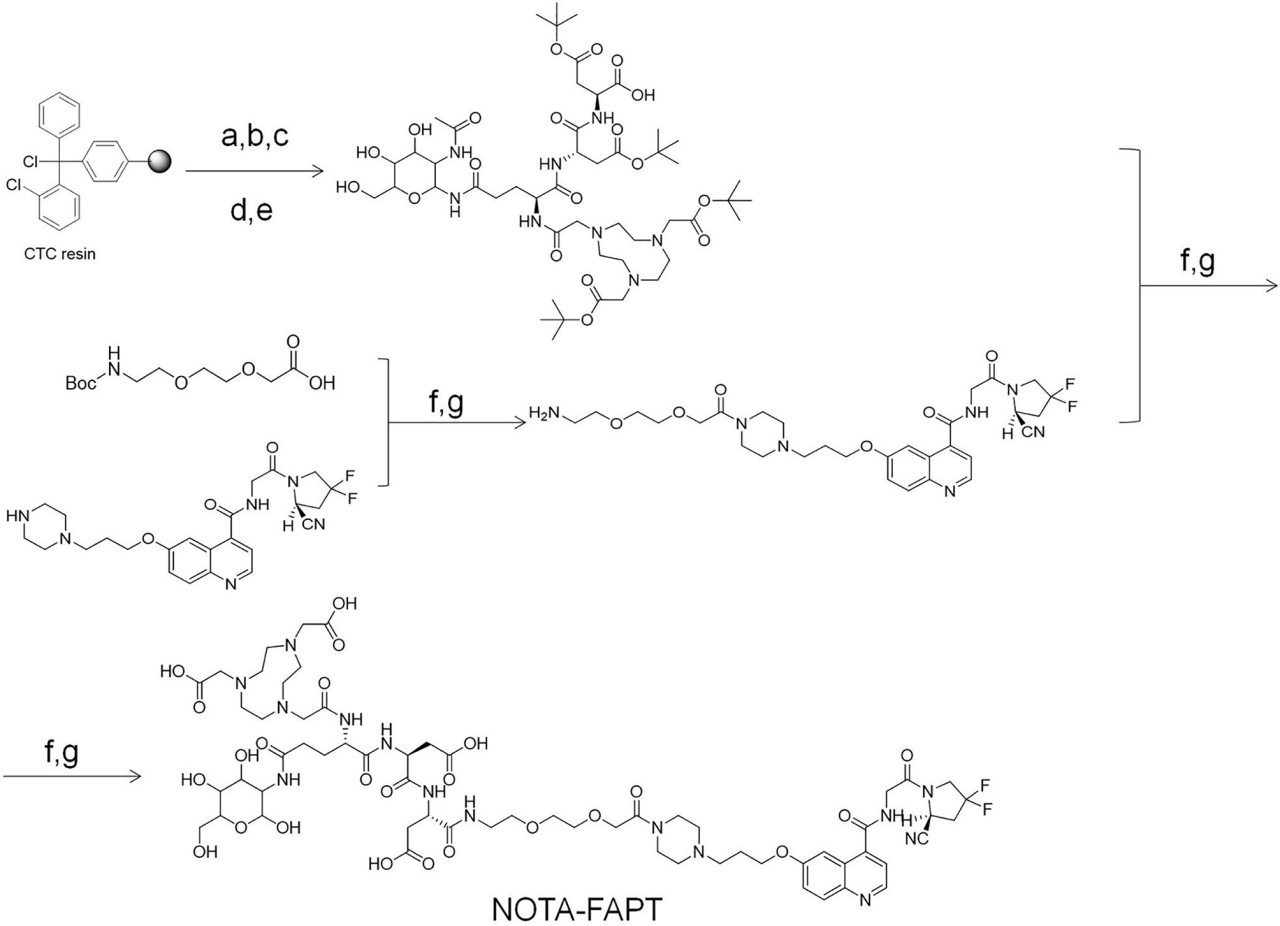
The specific synthesis process of precursors (NOTA-FAPT). Reagents and conditions: (a) Fmoc-Asp(OtBu)-OH; DIPEA; DCM; 3h 20% piperidine/DMF; (b) Fmoc-Asp(OtBu)-OH; DIC; HOBt; DMF 20% piperidine/DMF; (c) Fmoc-Glu (2-ACETAMIDO-2-DEOXY-BETA-D-GLUCOSAMINE)-OH; DIC; HOBt; DMF 20% piperidine/DMF; (d) NOTA; DIC; HOBt; DMF 20% piperidine/DMF; (e) 1%TFA/DCM; (f) HATU; DIPEA DMF; (g) TFA.

**Scheme 1** describes the specific synthesis process of precursors (NOTA-FAPT). The purity of the precursors was confirmed by Nanchang Tanzhen Biology (Nanchang, China), and their identity was determined through mass spectrometry. The [^19^F]AlF-FAPT was synthesized by mixing NOTA-FAPT (100 μg, 67.6 nmol), 34 μL of AlCl_3_ (2.0 mM; 68.0 nmol) in sodium acetate buffer (0.2 M; pH 4.0), 300 μL dimethyl sulfoxide (DMSO), and 300 μL of 0.5 M sodium acetate buffer (pH 4.0). A 30 μL solution of potassium fluoride (2.3 mM; 68.0 nmol) in water was then added, and the mixture was heated at 100°C for 30 min. The reaction mixture was finally purified using a Sep-Pak C18-plus cartridge (**Figures S1–S3**, and **Table S1**) (12).

### Automated Synthesis of [^18^F]AlF-FAPT

**Figure 1:**
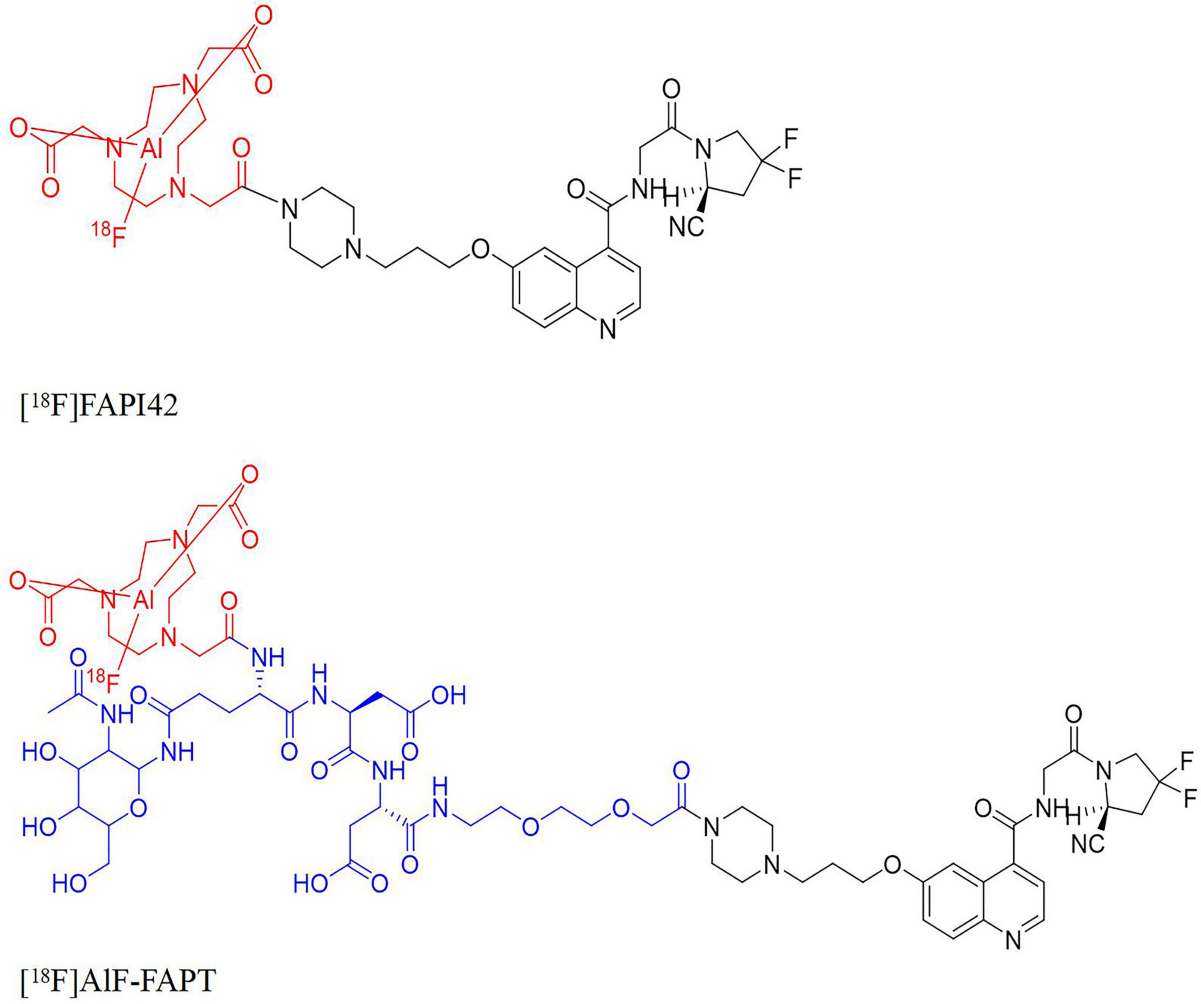
The structures of [^18^F]FAPI-42, [^18^F]AlF-FAPT, the red part was the NOTA ring chelated with aluminum fluoride and the blue part was the newly introduced group.

As previously reported, the automatic synthesis of [^18^F]AlF-FAPT was carried out on AllInOne (13–16). **Figure 1** and **Table 1** show the cassette layout, and the location of reagents and materials, respectively. In the process of installing the cassette, the reagent containing NOTA-FAPT (50 μg in 50 μl water), AlCl_3_(26.0 nmol; 13.0 μl; 2.0 mM) in sodium acetate buffer (pH 3.9; 0.2 M), and dimethylsulfoxide(DMSO, 300 μl) was added to the reactor and mixed well. The fluorine-18 (^18^F^-^) was transferred into the AllInOne module after being produced from the cyclotron and trapped in the QMA column (position H), which was pretreated with sodium acetate buffer (0.5 M; 5 ml) and water (10 ml). The ^18^F^-^ in the anion (QMA) column was then eluted into the reactor using sodium acetate buffer (300 μl; 0.5 M; pH 3.9; position A) and heated at 100°C for 15 min to complete the radiolabeling reaction. The mixture was then cooled to room temperature, followed by the addition of water (5 mL; position G) to the reactor, mixing, and transferring to the C18 column (position I), which was pretreated with anhydrous ethanol (10 ml) and water (10 ml). The C18 column was then rinsed thrice with 10 mL of water (position G) after transferring all the solutions to the reaction bottle. The product was finally eluted with 1.0 mL of ethanol: water at a ratio of 1:1 (v/v) (position E), filtered using a sterile filtration membrane, and collected in the receiving bottle. The product was then diluted into a solution containing 5% ethanol using normal saline (position F) for further studies.

**Table 1:** Positioning of reagents and materials on AllInOne module for the production of [^18^F]AlF-FAPT.

Position	Reagents or materials
A	^18^F- eluent (300 ul of 0.5 M pH 3.9 sodium acetate buffer)
B	Empty
C	ACN
D	Pipeline cleaning fluid (15mL ethanol)
E	product eluent (1.0 mL of ethanol and water in a ratio of 1:1(v/v))
F	Injection (10 mL of 0.9% NaCl)
G	Deionized water
H	^18^F^-^ separation cartridge (Sep-Pak light QMA)
I	product separation cartridge
Reactor	pre-added the mixture of AlCl3, precursor and DMSO

### Cell Studies and Animal Models

Human A549 cells (ATCC, USA) were grown at 37°C and 5% CO2 in DMEM (Gibco, USA) containing 10% fetal bovine serum (FBS, Gibco, USA) and 1% streptomycin and penicillin (Gibco, USA). The A549-FAP cells were then screened using 2 μg puromycin for two weeks and subsequently infected with lentivirus to stably express human FAP. A model of A549-FAP tumor-bearing nude mice was then established through subcutaneous tumorigenesis. All animal studies followed the guidelines of the Institutional Animal Care and Use Committee of Southern Medical University.

### Determination of Radiochemical Purity

The radiochemical purity of [^18^F]AlF-FAPT was confirmed by HPLC using a ZORBAX Eclipse XDB-C-18 analysis column. The HPLC included mobile phase A, a 0.1% acetonitrile solution of trifluoroacetic acid (TFA), and phase B, an aqueous solution of 0.1% TFA. The mobile phases were used to carry out a gradient elution. The ratio of phase A to phase B at 0 min was 10%:90% but gradually rose to 80%:20% at 10 min, which was maintained until the end of 15 min at a flow rate of 1mL/min. The elution curve was determined using an ultraviolet detector (Arminence SPD-M20A PDA, Shimadzu, Japan) and B-FC3200 high energy photomultiplier detector at 254 nm (Bioscan. Inc, Washington DC, USA). The non-radioactive standard [^19^F]AlF-FAPT was also injected into the HPLC to determine its retention time (Rt) and confirm the authenticity of the probe.

### Biological Distribution of [^18^F]AlF-FAPT

The biological distribution experiment of [^18^F]AlF-FAPT was carried out in normal Kunming mice. Twenty Kunming mice were randomly and equally divided into four groups and each injected with 0.1 ml of [^18^F]AlF-FAPT (1.48~2.22 MBq) through their tail vein. The mice were then sacrificed after 5, 30, 60, and 90 minutes, followed by weighing their organs and determining the radioactivity using a γ counter. The radioactivity of each organ was normalized using the percentage of injected dose per gram of tissue (% ID/g). [^18^F]FAPI-42 also used the same method mentioned above to carry out the biological distribution at the time point of 60 minutes.

### Micro-PET imaging and Immunohistochemical Staining

An Inveon Micro-PET/CT scanner was used to perform microPET dynamic imaging of tumor-bearing nude mice (A549 FAP; n = 4). The anesthetized tumor-bearing animals were placed in a prone position in a small animal PET scanner and intravenously injected with [^18^F]AlF-FAPT(8-10 MBq) and [^18^F]FAPI-42 (8-10 MBq). The dynamic microPET scans were then performed for 2 hours in tabular mode. PET data were collected at 5 min, 30 min, 60 min, 90 min, and 120 min after the animals were injected with [^18^F]AlF-FAPT through their tail veins. Low-dose CT scanning was used to perform attenuation correction (CTAC) and locate [^18^F]AlF-FAPT uptake sites in the micro-PET images. The three-dimensional ordered subset expected maximum (OSEM3D) algorithm (Siemens, Erlangen, Germany) was used for image reconstruction and conversion into a percentage of injected dose per gram of tissue (% ID/g). Data was analyzed by manually drawing the region of interest (ROI) above the tumor, followed by attenuation correction of the coronal image of the main organs on the whole body using the Inevon Research Workplace 4.2 software (Siemens, Erlangen, Germany).

Tumor tissues of A549-FAP tumor-bearing mice obtained by sacrificing the mice were immunohistochemically stained after preparation of paraffin-embedded sections using 4% paraformaldehyde. The sections were incubated with the FAP-alpha primary antibody (diluted 1:200, Affinity) and then with the goat anti-mouse secondary antibody (diluted 1:200, Servicebio). The secondary antibody was a molecule formed by combining horseradish peroxidase (HRP) and goat anti-mouse IgG. The sections were then reacted with DAB staining solution after combining the primary and secondary antibodies. DAB produced brown precipitation upon HRP catalysis, which amplified the signal and developed color. The immunohistochemical images were finally obtained after a series of routine processing.

### Statistical Analysis

Data were analyzed using the SPSS v22.0 software (IBM, Armonk, NY) and presented as means ± standard deviation (SD), which were then used to determine the differences between the two groups.

## Results

### Synthesis of Compounds

NOTA-FAPT precursors were designed and synthesized using solid resin and the standard FMOC solid-phase synthesis method (**Scheme 1**). The precursors and reference compounds of [^19^F]AlF-FAPT identified by HRMS had a chemical purity of > 95% (**Figure 3A**; **Supplementary Figures S1–S3**, and **Table S1**).

### Radiochemical Synthesis of [^18^F]AlF-FAPT

**Figure 2:**
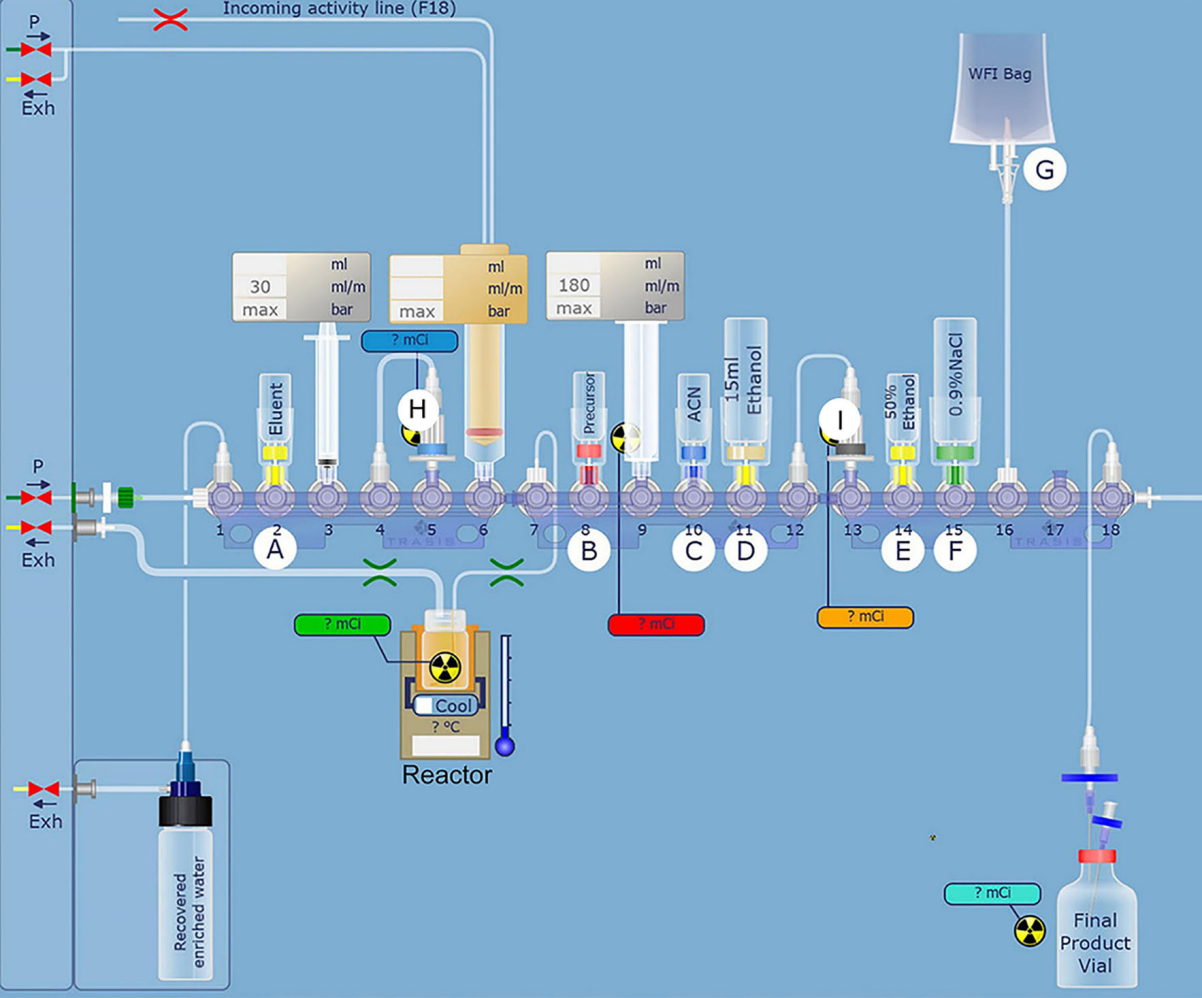
Automatic synthesis of [^18^F]AlF-FAPT using the AllInOne module.

**Figure 3:**
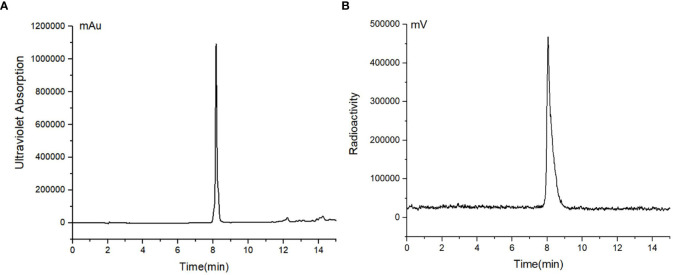
**(A)** HPLC chromatograms of standard unlabeled [^19^F]AlF-FAPT; **(B)** HPLC chromatograms of [^18^F]AlF-FAPT.

[^18^F]AlF-FAPT was successfully prepared using the automatic one-step procedure through the AllInOne module (**Figure 2**). The non-decay corrected radiochemical yield of [^18^F]AlF-FAPT was 25.4 ± 1.3% (n = 3) and the molar activity was 353.55 ± 82.25 GBq/μmol, with a radiochemical purity of more than 99% and a retention time of 8.38 min (**Figure 3B**). The total time of radiosynthesis, including radiolabeling and purification, was about 40min. The appearance of [^18^F]AlF-FAPT products had a colorless appearance free from particles, with a half-life of between 105 and 115 min. Both aerobes and anaerobes were negative after dilution of the product, with the bacterial endotoxin per 1mL being less than 15 EU and the ethanol content less than 5% (**Table 2**).

**Table 2:** Representative results of [^18^F]AlF-FAPT production using AllInone module.

Test	Specifications	AllInOne
Appearance	Colorless and free from particles	Colorless and free from particles
pH	5.0-8.0	5.9
Radio-chemical purity	≥90%	≥99%
Radioactive Concentration	≥370MBq/ml	≥370MBq/ml
RCY(%)	Not specified	25.4 ± 1.3
Residual organic solvent	Ethanol content<10%(v/v)	<5%
Residual organic solvent	DMSO ≤ 0.1%(v/v)	≤0.1%
Bacterial endotoxin	<17.5EU/ml	<15EU/ml
Aerobic bacteria and anaerobes detection	Negative	Negative
Radionuclidic identity	105-115mins	Meet the standard

### Biological Distribution of [^18^F]AlF-FAPT and [^18^F]FAPI-42

**Figure 4:**
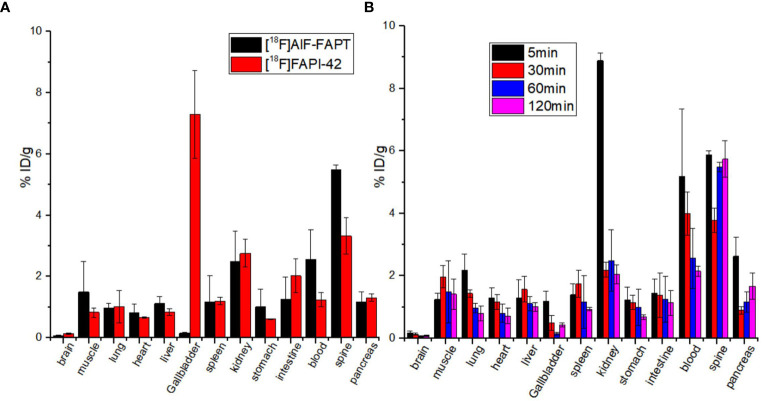
Biodistribution study of [^18^F]AlF-FAPT and [^18^F]FAPI-42 at different tissues. **(A)** The biodistribution of [^18^F]AlF-FAPT and [^18^F]FAPI-42 at 60 minutes; **(B)** The biodistribution of [^18^F]AlF-FAPT at different time points.

**Figure 4A** shows the results of biodistribution of [^18^F]AlF-FAPT and [^18^F]FAPI-42 in Kunming mice. Compared with [^18^F]FAPI-42 in the 60 min, the radioactivity uptake value of [^18^F]AlF-FAPT in the gallbladder and was lower. Especially in the gallbladder, the uptake value of [^18^F]FAPI-42 could go up to 7.30 ± 1.43%ID/g, while the uptake value of [^18^F]AlF-FAPT was only 0.55 ± 0.18%ID/g. [^18^F]AlF-FAPT showed relatively high uptake values in the muscle and blood. Both of them showed relatively high uptake values in the spine. **Figure 4B** shows the radioactivity uptake in the kidney was high, with an uptake value of 8.90 ± 0.23%ID/g at 5 min and descended rapidly to 2.19 ± 0.24%ID/g at 30 min. These findings suggested that [^18^F]AlF-FAPT was primarily metabolized through the kidney pathway and less through the hepatobiliary pathway. Notably, there was a relatively low radioactivity uptake and rapid radioactivity clearance in other organs such as the stomach, spleen, lung, heart, brain, muscles, and intestines. The radioactivity levels of these organs were positively correlated with the corresponding PET imaging and were beneficial to the prominent uptake of tumors.

### Micro-PET Imaging and Immunohistochemical Staining

**Figure 5:**
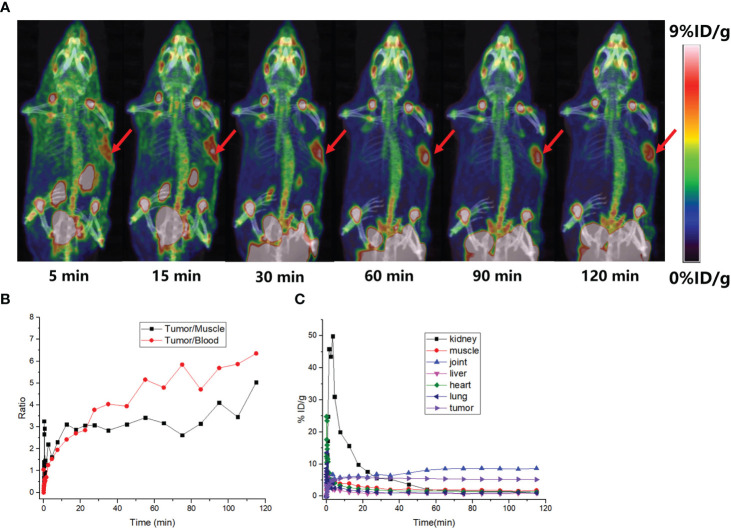
PET imaging of small animals. **(A)** MIP diagrams at different time points, the red arrows points to the tumor; **(B)** The black curve is the ratio of tumor-muscle with time and the red curve is the ratio of tumor-blood with time; **(C)** The time-dependent uptake curves of different organs in the body.

**Figure 6:**
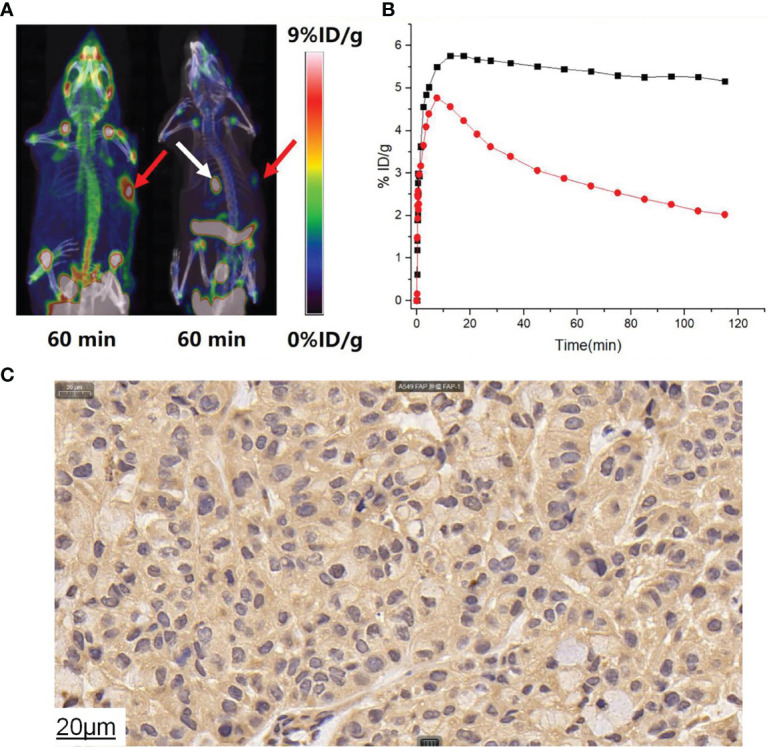
**(A)** [^18^F]AlF-FAPT and [^18^F]FAPI-42 took contrast images during 60min, the red arrows points to the tumor and the white arrow points to the gallbladder. **(B)** The time-dependent uptake curves of [^18^F]AlF-FAPT (black) and [^18^F]FAPI-42 (white) in the tumor. **(C)** IHC staining results showed A549FAP tumor tissue with high FAP expressing (40× amplification).

Typical micro-PET/CT images (MIP images) were obtained at six different time points after injecting tumor-bearing nude mice (A549 FAP human lung adenocarcinoma mouse model) with [^18^F]AlF-FAPT (**Figure 5A**). The higher radioactivity uptake in the kidneys and bladder suggested that the urinary system (kidney-bladder) was the primary excretory route for the tracer. PET studies also showed that [^18^F]AlF-FAPT has a high tracer uptake in the spine, which was consistent with the previously mentioned biological distribution. Notably, [^18^F]AlF-FAPT uptake in A549-FAP tumors peaked at 10 min and remained stable until 120 min. The tumor had a good contrast with the background in the mouse model because of the low uptake in the lungs, muscles, gastrointestinal tract, and brain (**Figures 5A, C**). The tumor-blood and tumor-muscle ratio stabilized at 60 min and remained that way until 120 min, suggesting that 60 min was the best imaging point for [^18^F]AlF-FAPT. This time point was employed during the follow-up contrast imaging with [^18^F]FAPI-42 (**Figure 5B**). In the same A549-FAP tumor model, the uptake and retention of the two imaging agents in the tumor were directly showed from the curve of tumor uptake value with time. Though both tracers reached high uptake values within a short period, [^18^F]AlF-FAPT uptake remained stable up to 120 min, while [^18^F]FAPI-42 decreased gradually with time to a final uptake value of about 2%ID/g. These findings suggested that [^18^F]AlF-FAPT was superior to [^18^F]FAPI-42 in tumor uptake (**Figure 6B**). What’s more, [^18^F]FAPI-42 exhibited a higher uptake than [^18^F]AlF-FAPT in the gallbladder and intestines uptake. The background uptake value of [^18^F]AlF-FAPT was higher than that of [^18^F]FAPI-42 based on the context of the same %ID/g value (**Figure 6A**). IHC results further revealed that the tumor developed in the A549-FAP lung adenocarcinoma model had a high expression of FAP (**Figure 6C**).

## Discussion

This study reports on the automatic synthesis, biodistribution, and imaging results of a new ^18^F-labelled FAP tracer, [^18^F]AlF-FAPT, and directly compares it with tracer [^18^F]FAPI-42.

Routine clinical production of PET-radiopharmaceuticals require one batch to produce enough products to achieve a high batch activity, meet the needs of multiple patients and reduce cost. Automatic synthesis produces high batch activity of ^18^F-labeled radiopharmaceuticals, thus reducing radiation exposure to production operators. The Al^18^F method developed by McBride et al. makes it possible to label peptides and proteins directly using radionuclide ^18^F^-^ (17). In this study, [^18^F]AlF-FAPT was successfully synthesized by Al^18^F chelation reaction using the Trasis AllInOne module. In contrast to the GETRACERlabFX2N module, which reuses the reaction bottle, the cassette-based AllInOne module replaces the cassette after one or two reactions, thus reducing the contamination of metal ions and enhancing the success of the reaction (13). The quality control analysis results of the developed product were consistent with all the accepted standards of the product (**Table 2**).

The carbohydration has been proven to improve general pharmacokinetics, which could significantly increase the kidney clearance and reduced hepatobiliary excretion (18). Previous studies postulate that introducing a PEG linker can increase the metabolic stability and *in vivo* half-life of the products (19, 20). The introduction of hydrophilic linkers such as PEG and amino acids such as Asp and Glu to the radiotracer [^18^F]AlF-FAPT could increase the hydrophilicity and reduce the lipophilicity of [^18^F]AlF-FAPT, which contributed to the reduction of hepatobiliary metabolism of [^18^F]AlF-FAPT. The dynamic Micro-PET imaging of A549-FAP xenografts demonstrated that [^18^F]AlF-FAPT can specifically bind to the tumor, thus having a significantly higher tumor uptake than [^18^F]FAPI-42. Though the uptake of both tracers reached the highest value at about 10 min, the [^18^F]AlF-FAPT probe remained stable in the tumor until 120 min (5.16 ± 1.65%ID/g), while the [^18^F]FAPI-42 gradually decreased to an uptake value of 2.02 ± 0.81% ID/g at 120 min. Despite the uptake of the [^18^F]FGlc-FAPI tracer designed by Toms J et al. being stable, its significant non-specific uptake in the liver and intestines poses a challenge in detecting the abdominal FAP-positive lesions in cancer patients (21). Also, in the contrast imaging and biodistribution results of [^18^F]AlF-FAPT and [^18^F]FAPI-42 revealed that [^18^F]FAPI-42 had a significant high uptake in the gallbladder, which was consistent with the findings of previous studies (11). Notably, the uptake of [^18^F]AlF-FAPT in the gallbladder and liver was significantly lower, suggesting that it was not metabolized through the hepatobiliary pathway. This finding was beneficial to the imaging of abdominal tumors. Interestingly, we found high spinal uptake in both [^18^F]AlF-FAPT and [^18^F]FAPI-42. The biological distribution of [^18^F]FGlc-FAPI displayed the same high uptake in spine and almost no uptake in spine when blocking. Thus, we assumed that fibroblast activation protein (FAP) is highly expression in spine so the radiotracers targeted FAP display high uptake in spine. Moreover, [^18^F]AlF-FAPT exhibited a higher uptake at the joint, which was consistent with the previously reported ^18^F-labeled FAPI (11, 21, 22). Collectively, [^18^F]AlF-FAPT is a better FAPI imaging agent than previously reported ^18^F-labeled FAPI.

In conclusion, we have clearly proves the feasibility of using [^18^F]AlF-FAPT as a new radioactive tracer for PET imaging.

## Data Availability Statement

The original contributions presented in the study are included in the article/**Supplementary Material**. Further inquiries can be directed to the corresponding authors.

## Ethics Statement

The animal study was reviewed and approved by the Institutional Animal Care and Use Committee of Southern Medical University.

## Author Contributions

Study conception and design, GT and YHH. Acquiring data, JH, KH, SH, YJH, LF, RL, and WX. Analysis of data, JH, KH, and SH. Drafting the manuscript, JH. Revising the manuscript, GT and YHH. All authors contributed to the article and approved the submitted version.

## Funding

This work is supported by the National Natural Science Foundation of China (91949121), and the Talent introduction Fund of Nanfang Hospital of Southern Medical University (123456, China).

## Conflict of Interest

The authors declare that the research was conducted in the absence of any commercial or financial relationships that could be construed as a potential conflict of interest.

## Publisher’s Note

All claims expressed in this article are solely those of the authors and do not necessarily represent those of their affiliated organizations, or those of the publisher, the editors and the reviewers. Any product that may be evaluated in this article, or claim that may be made by its manufacturer, is not guaranteed or endorsed by the publisher.
