# Supplementary material for: Automatic Production and Preliminary PET Imaging of a New Imaging Agent [18F]AlF-FAPT
Source: Front Oncol. 2022 Jan 6;11:802676. doi: 10.3389/fonc.2021.802676 (PMC8770261; doi:10.3389/fonc.2021.802676)
Supplement: Supplementary file 1 [file DataSheet_1.docx]

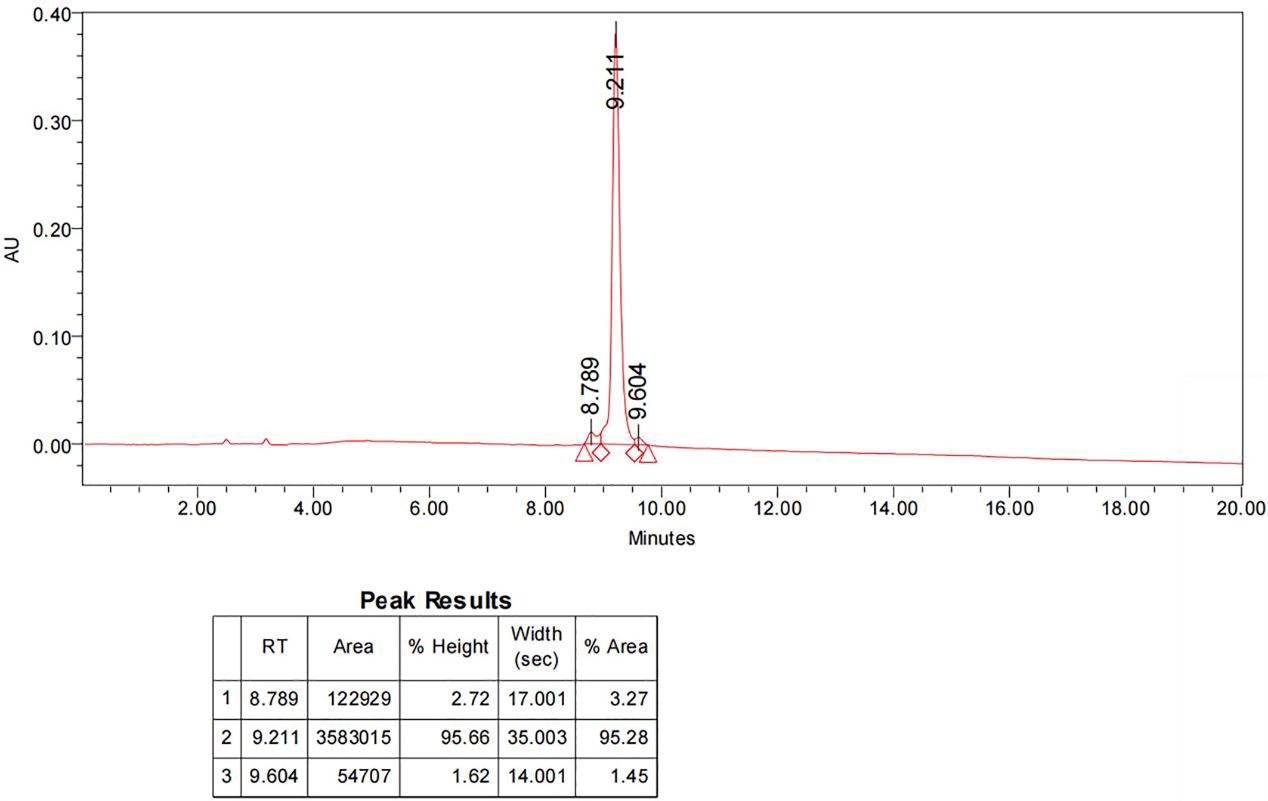


Figure S1.The HPLC chromatograms of precursors(NOTA-FAPT)


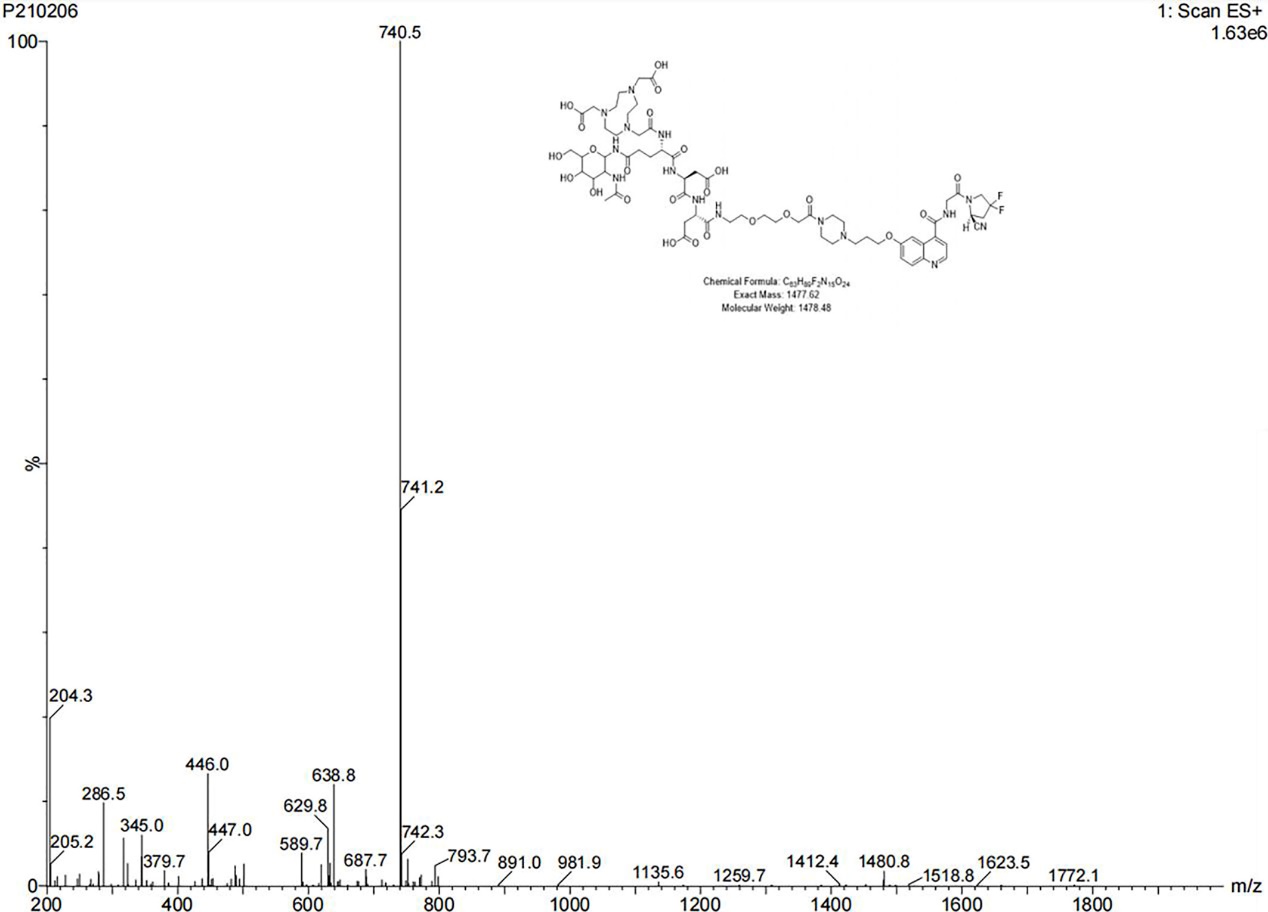


Figure S2. The mass spectrometry of precursors(NOTA-FAPT)

Table S1.The quality control of precursors(NOTA-FAPT)

| **Product Name:** | FAPI-2-ACETAMIDO-2-DEOXY-BETA-D-GLUCOSAMINE |
| --- | --- |
| **Molecular Weight:** | 1478.48 |
| **Mass Spectral Analysis:**  **Column:**  **Mobile phase:** | see attached MS spectrogram  Sepax GP-C18 5um 120A 4.6*250mm  A:0.1%TFA in 100%H2O B:0.1%TFA in 100%ACN |
| **HPLC Analysis:** | Peptide purity:>95% |
| **Solubility:** | N/A |
| **Appearance:** | White lyophilized powder |
| **Counter Ion:** | N/A |


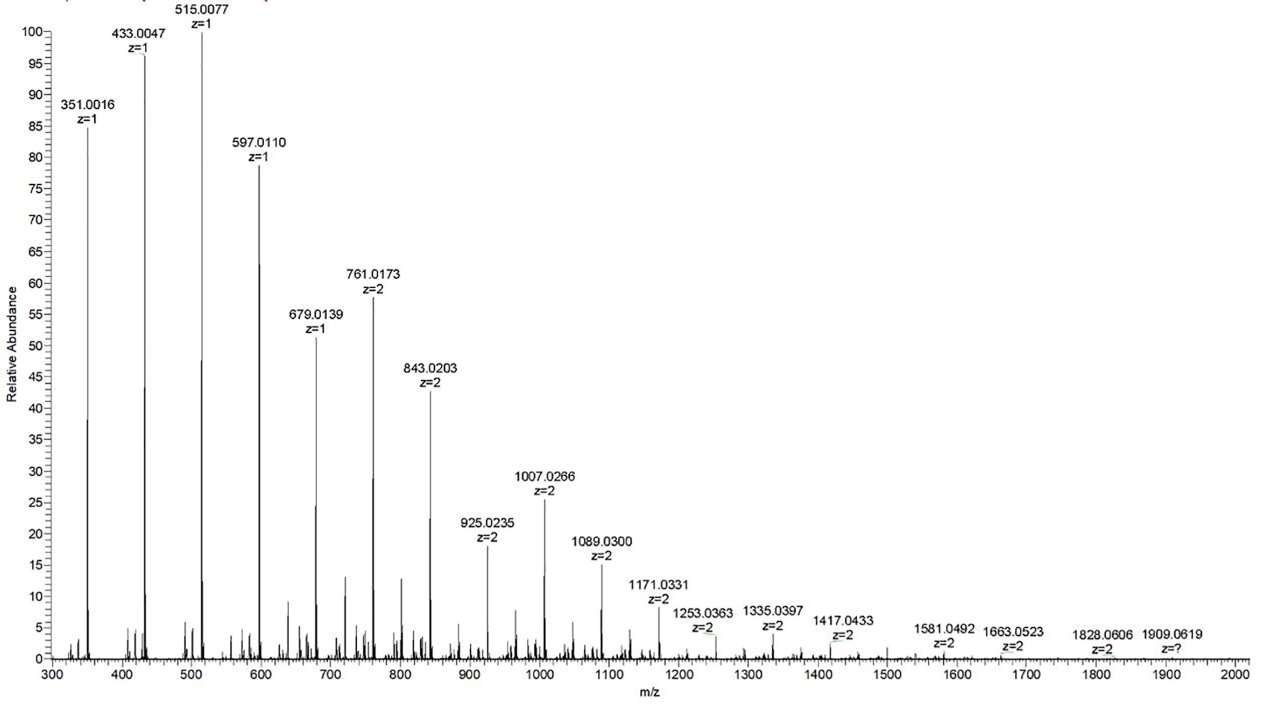


Figure S3. The High-resolution mass spectrometry of [^19^F]AlF-FAPT.
